# Fissures in Ano Treated with Iodoform

**Published:** 1874-05

**Authors:** 


Fissures in Ano Treated with Iodoform.—Dr. Francesco Parona (*Giornale Italiano delle Mal. Ven. e della Pelle,* October,) gives his experience of iodoform in fissures of the anus, and strongly recommends it. He believes it acts in great measures as a local anæsthetic, "which allays the spasm of the sphincter during defæcation, while it favors cicatrisation by neutralizing the irritating effects of fœcal matters which may remain on the ulcerated surface." It has also a direct healing action. Dr. Parona uses the iodoform as an ointment (one part to three of lard), and applies it on a small cylinder of charpie, of a size requiring but little force for its introduction. The charpie has the advantage that its filaments adapt themselves readily to the slight irregularities of the anal mucous membrane. The dressing is changed twice a day, and replaced after each motion, and in the majority of cases the pain and spasm caused by the fissure cease in a few days, and the patient is well in a relatively short time. Of four cases, of which details are given, the longest time was twenty days, while one of the patients had been ill four months previous to the treatment.—*Medical Times and Gazette.*

